# Altered Whole-Brain and Network-Based Functional Connectivity in Parkinson's Disease

**DOI:** 10.3389/fneur.2018.00419

**Published:** 2018-06-06

**Authors:** Laura J. de Schipper, Anne Hafkemeijer, Jeroen van der Grond, Johan Marinus, Johanna M. L. Henselmans, Jacobus J. van Hilten

**Affiliations:** ^1^Department of Neurology, Leiden University Medical Center, Leiden, Netherlands; ^2^Department of Radiology, Leiden University Medical Center, Leiden, Netherlands; ^3^Department of Methodology and Statistics, Institute of Psychology, Leiden University, Leiden, Netherlands; ^4^Leiden Institute for Brain and Cognition, Leiden University, Leiden, Netherlands; ^5^Department of Neurology, Antonius Hospital, Woerden, Netherlands

**Keywords:** Parkinson's disease, resting-state, functional magnetic resonance imaging, eigenvector centrality mapping, network, connectome

## Abstract

**Background:** Functional imaging methods, such as resting-state functional magnetic resonance imaging, reflect changes in neural connectivity and may help to assess the widespread consequences of disease-specific network changes in Parkinson's disease. In this study we used a relatively new graph analysis approach in functional imaging: eigenvector centrality mapping. This model-free method, applied to all voxels in the brain, identifies prominent regions in the brain network hierarchy and detects localized differences between patient populations. In other neurological disorders, eigenvector centrality mapping has been linked to changes in functional connectivity in certain nodes of brain networks.

**Objectives:** Examining changes in functional brain connectivity architecture on a whole brain and network level in patients with Parkinson's disease.

**Methods:** Whole brain resting-state functional architecture was studied with a recently introduced graph analysis approach (eigenvector centrality mapping). Functional connectivity was further investigated in relation to eight known resting-state networks. Cross-sectional analyses included group comparison of functional connectivity measures of Parkinson's disease patients (*n* = 107) with control subjects (*n* = 58) and correlations with clinical data, including motor and cognitive impairment and a composite measure of predominantly non-dopaminergic symptoms.

**Results:** Eigenvector centrality mapping revealed that frontoparietal regions were more prominent in the whole-brain network function in patients compared to control subjects, while frontal and occipital brain areas were less prominent in patients. Using standard resting-state networks, we found predominantly increased functional connectivity, namely within sensorimotor system and visual networks in patients. Regional group differences in functional connectivity of both techniques between patients and control subjects partly overlapped for highly connected posterior brain regions, in particular in the posterior cingulate cortex and precuneus. Clinico-functional imaging relations were not found.

**Conclusions:** Changes on the level of functional brain connectivity architecture might provide a different perspective of pathological consequences of Parkinson's disease. The involvement of specific, highly connected (hub) brain regions may influence whole brain functional network architecture in Parkinson's disease.

## Introduction

Parkinson's disease (PD) is characterized by a broad spectrum of motor and non-motor symptoms, which are linked to a progressive formation of α-synuclein (α-SynA) aggregates in presynaptic terminals, Lewy neurites and Lewy bodies in neurons of the central and peripheral nervous system (1, 2). α-SynA are not randomly distributed in the brain, but appear in select regions, which likely are affected because of shared anatomical and functional properties among neurons (3, 4). Depositions are most distinct in the midbrain, pontine and medullary nuclei and limbic structures, but are also found in the neocortex in the more advanced stages of the disease (1, 4). Compelling evidence shows that α-SynA-related synaptic dysfunction antedates nerve cell loss, suggesting that altered neuronal connectivity is a key feature in PD (5).

Functional imaging methods, such as resting-state functional magnetic resonance imaging (fMRI), reflect changes in neural connectivity and may help to assess the widespread consequences of disease-specific network changes in neurodegenerative diseases (6). Former fMRI studies indicate decreases as well as increases in functional connectivity in PD patients (7, 8). Most of these studies focused on functional connectivity of (multiple) brain regions or networks of interest, thus precluding inferences on a whole-brain level of integrated networks that are spatially distributed, but functionally linked.

A relatively new graph analysis approach in functional imaging concerns eigenvector centrality mapping (ECM). ECM identifies prominent regions in the brain network hierarchy and detects localized differences between patient populations (9). Since this model-free method is applied to all voxels in the brain it does not require a priori selection of potentially involved networks and is not restricted to one area (regions of interest) of the brain. The main difference between the method used in our study and other graph analysis studies in PD is that our approach counts the neighbors of each vertex, weighted by their centralities (9). ECM has been linked to changes in functional connectivity in certain nodes of brain networks that might contribute to depression in patients with PD (10). Changes in ECM have also been linked to cognition in APOE ε4 carriers (11), but also to neurodegenerative changes in type 1 diabetes mellitus (12), and cognitive dysfunction and physical disability in multiple sclerosis (13).

The aim of this study was to evaluate and compare the consequences of PD on the resting-state functional connectivity on a whole-brain level of integrated networks and on eight explicit brain networks. We hypothesized that these combined approaches can identify regionally specific differences in functional connectivity of the brain between PD patients and control subjects.

## Patients and methods

### Study design and participants

The present cross-sectional study in PD patients is part of the PROfiling Parkinson's disease (PROPARK) study. PD patients were recruited from the outpatient clinic for Movement Disorders of the Department of Neurology of the Leiden University Medical Center (LUMC; Leiden, the Netherlands) and nearby university and regional hospitals. All participants fulfilled the United Kingdom Parkinson's Disease Society Brain Bank criteria for idiopathic Parkinson's disease (14). Evaluations occurred between January 2013 and 2016. Exclusion criteria were: previous or other disorders of the central nervous system, peripheral nerve disorders influencing motor and/or autonomic functioning, and psychiatric comorbidity not related to PD. Patients were matched at group level for age and gender with a group of healthy control subjects. Control subjects were recruited from the Leiden Longevity Study (LLS), a study set up to identify genetic and phenotypic determinants of longevity in healthy long-living families (15).

### Clinical assessments

All patients underwent standardized assessments, including an evaluation of demographic and clinical characteristics. Almost all patients, except for 13 individuals (twelve *de novo* patients, defined as dopaminergic drug-naïve patients with a disease duration shorter than 5 years; one other dopaminergic drug-naïve patient), were tested while on dopaminergic medication. The Movement Disorder Society Unified Parkinson's Disease Rating Scale (MDS-UPDRS) motor scale (part III) was used to quantify the severity of motor symptoms (16). Additionally, the SEverity of Non-dopaminergic Symptoms in Parkinson's disease (SENS-PD) scale was used, which is a composite score comprising three items with four response options (0–3) from each of the following six domains: postural instability and gait difficulty, psychotic symptoms, excessive daytime sleepiness, autonomic dysfunction, cognitive impairment and depressive symptoms (total range: 0–54) (17). These six domains represent a coherent complex of symptoms that largely do not improve with dopaminergic medication, that is already present in the early disease stages, and increases in severity when the disease advances (17). Higher scores on both scales reflect more severe impairment. The SCales for Outcomes in PArkinson's disease-COGnition (SCOPA-COG; cognitive functioning; range 0–43) was used to assess cognitive performance. The SCOPA-COG is a valid and reliable instrument examining the following domains: memory, attention, executive functioning and visuospatial functioning (18); lower scores reflect more severe impairment. A levodopa dose equivalent (LDE) of daily levodopa (LDE-Dopa), dopamine agonists (LDE-DA), as well as a total LDE was calculated according to the formula developed by Tomlinson et al. (19).

### MRI acquisition

Imaging was performed on a 3 Tesla MRI scanner (Philips Achieva, Best, the Netherlands). Resting-state fMRI images were acquired with the following parameters: repetition time = 2.2 s, echo time = 30 ms, flip angle = 80°, 37 slices, resulting in a voxel size of 2.75 × 2.75 × 2.72 mm, with a 10% interslice gap, 200 volumes, scan duration 7 min and 29 s. Participants were instructed to lie still with their eyes closed and not to fall asleep during the scan. For registration purposes three-dimensional T1-weighted anatomical images were acquired with the following parameters: repetition time = 9.8 ms, echo time = 4.6 ms, flip angle = 8°, FOV 220 × 174 × 156 mm, 130 slices with a slice thickness of 1.2 mm with no gap between slices, resulting in a voxel size of 1.15 × 1.15 × 1.20 mm. Additionally, a high-resolution echo planar image was obtained with the following parameters: repetition time = 2.2 s, echo time = 30 ms, flip angle = 80°, 84 slices, resulting in a voxel size of 1.96 × 1.96 × 2.00 mm with no gap between slices.

### Data analysis

Before analysis, all MRI scans were visually checked to ensure that no major artifacts or abnormalities were present in the data. Analyses were done using the software provided using the FMRIB's software library (FSL; version 5.0.8, Oxford, United Kingdom) and Matlab software (version 6.1, The MathWorks Inc., Natick, MA, 2000) (20).

#### Preprocessing

The preprocessing of the resting-state fMRI data consisted of motion correction using motion correction FMRIB's Linear Image Registration Tool (MCFLIRT) (21), brain extraction (22), spatial smoothing using a Gaussian kernel with a full width at half maximum of 5 mm, high-pass temporal filtering with a cutoff frequency 0.01 Hz, non-linear registration with Boundary-Based Registration to the 2 mm isotropic Montreal Neurological Institute −152 standard space image (MNI; Montreal, QC, Canada) and a 10 mm warp resolution (23), via the T1-weighted images, using high-resolution echo planar images for an additional registration step between functional images and T1-weighted images (24). ICA-based Automatic Removal Of Motion Artifacts (ICA-AROMA) was used to identify and remove residual motion-related artifacts from the resting-state data (25, 26).

#### Eigenvector centrality mapping

ECM was used to identify prominent nodes in the whole brain network per group. Localized differences between PD patients and control subjects were subsequently calculated. Each voxel is assigned its own value of relevance to the network (27). Higher EC indicates a more prominent role in the brain network hierarchy and lower EC a less prominent role.

For each participant, we calculated a whole brain eigenvector centrality map in standard space, using the fast ECM algorithm (9, 28). The EC maps of all participants (i.e., PD patients and control subjects) were concatenated into a single four-dimensional data set. A gray matter mask was applied to make sure that only gray matter centrality was studied. FSL-Randomise permutation-testing tool for nonparametric permutation inference was used for statistical analysis, with 5000 permutations (29). Group differences in mean EC values were calculated using a two-sample *T*-test. The Threshold-Free Cluster Enhancement (TFCE) technique was used to correct for multiple comparisons across space. Statistical threshold was set at *p* < 0.05, Family-Wise Error (FWE) corrected, applying a minimum cluster size of 40 mm^3^ (30).

#### Resting-state networks

Eight standard resting-state networks were used as a template to study whole-brain functional connectivity in a standardized way: (1) medial visual network; (2) lateral visual network; (3) auditory system network; (4) sensorimotor system network; (5) default mode network; (6) executive control network; (7 and 8) dorsal visual stream networks (31, 32). Resting-state functional connectivity was studied using the dual regression method of FSL (33), according to Hafkemeijer et al. (32). In short, this method results in 10 3D images per individual (eight resting-state networks and a white matter and cerebrospinal fluid template to further account for noise) with voxel-wise z-scores representing the functional connectivity in each of the templates (32). Voxel-wise group differences between spatial maps of PD patients and control subjects were tested using FSL-Randomise using the same design as in the ECM analysis, TFCE-FWE corrected and a statistical threshold of *p* < 0.05 (29, 30). The standard resting-state networks were used as masks in the analyses.

### Statistical analysis

The following statistics were performed in SPSS (IBM SPSS Statistics for Mac, Version 23.0. Armonk, NY: IBM Corp.). Differences in demographic data between patients and control subjects were analyzed with an independent-sample *T*-test (age) and a chi-square test (gender). For brain areas with significant group differences in EC, mean EC values were extracted. Z-scores were extracted from brain areas with significant group differences in resting-state functional connectivity networks. Within the PD group, relationships between mean functional connectivity measures (i.e., mean EC values and z-scores; dependent variable) and the MDS-UPDRS motor score, SENS-PD score and SCOPA-COG were studied using a general linear model. Age, gender and gray matter volume, normalized for subject head size as estimated with Structural Image Evaluation using Normalization of Atrophy Cross-sectional (SIENAX) (20), were included as covariates in the model (34). Relationships were subsequently studied within the group of PD patients who used dopaminergic medication, while adjusting for the total LDE. Bonferroni correction was applied to account for multiple comparisons.

## Results

### Demographic characteristics

In total, one hundred and sixty-five subjects, of which one hundred and seven patients and fifty-eight control subjects were included in the analysis. Demographic and clinical data of all participants are shown in Table 1. There were no significant differences in age (*p* = 0.605) and gender (*p* = 0.206) between the patient and the control group.

**Table 1:** Main characteristics of participants.

**Characteristic (score range)**	**Patients**	**Controls**
**N**	**107**	**58**
Men/women (% men)	68/39 (63.6)	31/27 (53.4)
Age, years	64.6 (6.9)	65.2 (7.5)
Disease duration, years	9.5 (4.8)	n/a
MDS-UPDRS motor score (0–132)	33.2 (15.5)	n/a
SENS-PD, *n* = 99 (0–54)	13.5 (6.1)	n/a
SCOPA-COG, *n* = 100 (0–43)	27.5 (5.8)	n/a
Total LDE, mg/day, *n* = 92	995.6 (550.4)	n/a
Drug-naïve patients, n = 1	n/a	n/a
*De novo* patients^*^, *n* = 12	n/a	n/a
LDE-Dopa, mg/day, *n* = 92	739.8 (486.4)	n/a
LDE-DA dose, mg/day, *n* = 92	184.6 (208.2)	n/a

Values are means (standard deviation) for continuous variables and numbers for dichotomous variables. MDS-UPDRS: Movement Disorder Society-Unified Parkinson's Disease Rating Scale; SENS-PD: SEverity of Non-dopaminergic Symptoms in Parkinson's Disease; SCOPA-COG: SCOPA cognition; LDE: Levodopa dosage equivalent; DA: dopamine agonists; n/a: not applicable.

* *De novo patients: drug-naïve patients with disease duration shorter than 5 years*.

### Eigenvector centrality maps

#### Mean eigenvector centrality maps

The mean EC group map of control subjects and the mean EC group map of PD patients both showed highest cortical mean EC values in the precuneus, posterior cingulate gyrus and occipital lobe. In the control group, highest subcortical mean EC values were found in the thalamus, hippocampus and caudate nucleus. In the patient group, highest subcortical mean EC values in patients were found in the thalamus and hippocampus.

#### Patient–control subject comparisons (Figure 1)

**Figure 1:**
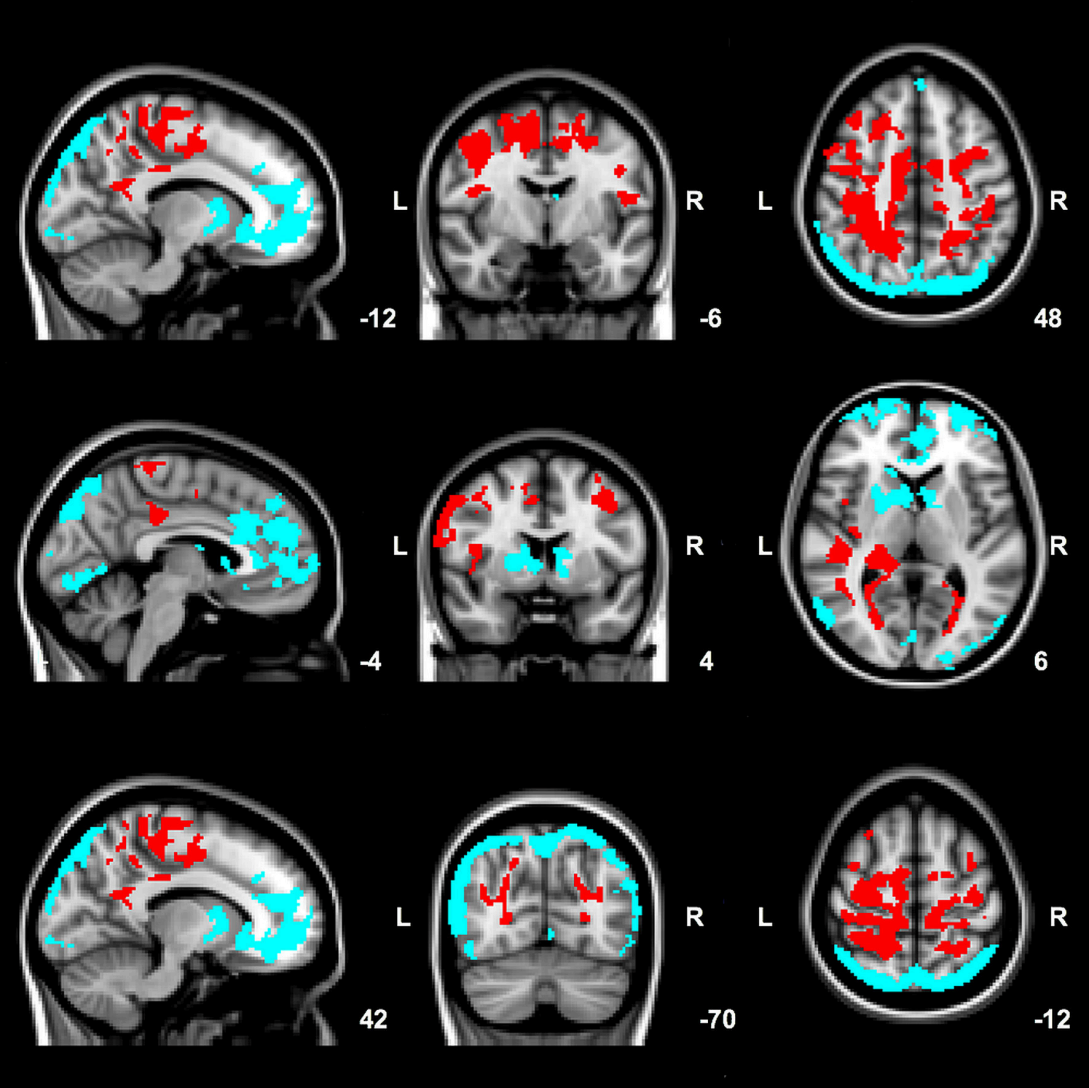
Significant group differences between patients and control subjects in mean EC values. Red: clusters of increased EC in patients compared to control subjects. Blue: clusters of decreased EC in patients compared to control subjects. Results are corrected for age, gender and voxel-wise gray matter volume per subject. Images are overlaid on the most informative, sagittal, coronal, and axial slices of the MNI standard anatomical image (MNI coordinates of each slice are given).

Compared to control subjects, PD patients had increases in EC in large areas of the parietal and frontal lobe (opercular cortex, superior division of the lateral occipital cortex, posterior cingulate gyrus, precuneus, superior parietal lobe, supplementary motor area (SMA), pre- and postcentral gyrus, frontal gyrus and right middle frontal gyrus), right temporal lobe, right thalamus and a small region of the right hippocampus (Figure 1, red areas).

Further, compared to control subjects, patients had areas of decreased EC in the occipital lobe (cuneus, intracalcarine cortex, lingual gyrus, occipital pole), frontal lobe (frontal gyrus, frontal medial cortex, anterior cingulate gyrus, paracingulate gyrus, and frontal pole), inferior division of the lateral occipital cortex, precuneus and right pallidum and anterior putamen (Figure 1).

We found no significant relations between mean EC values of brain areas with group differences and the MDS-UPDRS motor score, SENS-PD score, or SCOPA-COG score.

### Resting-state network analyses

**Figure 2:**
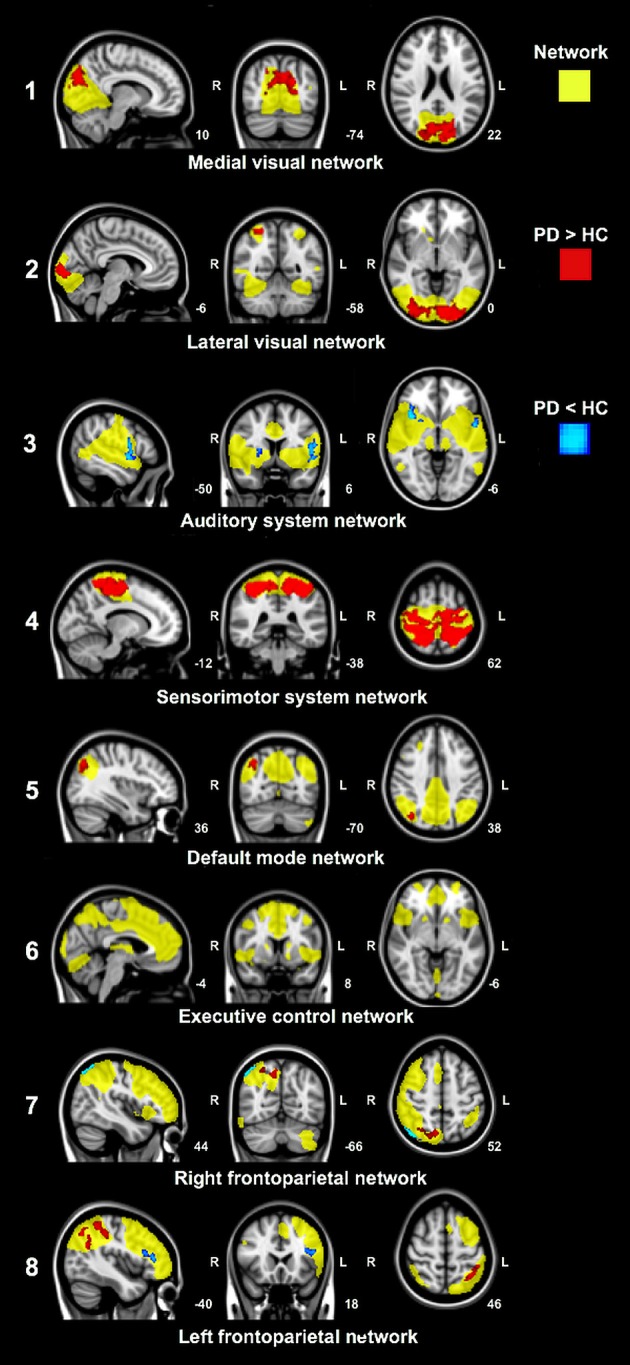
Significant group differences in functional connectivity between Parkinson's disease patients and control subjects in resting-state networks. Analyses were adjusted for age, gender and voxel-wise gray matter volume per subject. Yellow: Spatial maps of eight standard resting-state networks. Increased (red) and decreased (blue) network connectivity in patients compared to control subjects. Images are overlaid on the most informative, sagittal, coronal, and axial slices of the MNI standard anatomical image (MNI coordinates of each slice are given).

Voxel-wise group comparisons of the resting-state network connectivity revealed group differences in functional connectivity between PD patients and control subjects in seven out of eight resting-state networks (Figure 2). Group differences were most pronounced in the medial and lateral visual network and the sensorimotor system network (Figure 2, network 1, 2, and 4). Increased functional connectivity in PD patients compared to control subjects was found between the cuneus, precuneus, superior division of the lateral occipital cortex, intra- and supracalcarine cortex and lingual gyrus, and the medial visual network; between the superior parietal lobe, superior division of the lateral occipital cortex, lingual gyrus, occipital pole and the lateral visual network; and between the superior parietal lobe, SMA, pre- and postcentral gyrus, precuneus, cingulate gyrus, supramarginal gyrus and the sensorimotor system network (Figure 2, network 1, 2, and 4). Smaller regions of increased connectivity were found between the precuneus, superior division of the lateral occipital cortex and the default mode network (DMN) and right frontoparietal network (Figure 2, network 5 and 7); and between the superior division of the lateral occipital cortex, angular gyrus, supramarginal gyrus, subcallosal cortex, superior parietal lobe and the left frontoparietal network (Figure 2, network 8).

Decreased functional connectivity in PD patients compared to control subjects was found between the insular cortex, opercular cortex, frontal orbital cortex, precentral cortex, right putamen and the auditory system network; between the superior division of the lateral occipital cortex and the right frontoparietal network; and between the frontal orbital cortex and frontal gyrus and the left frontoparietal network (Figure 2, network 3, 7, and 8). No changes in functional connectivity were observed in the executive control network (Figure 2, network 6).

We found no significant relations between mean z-scores of regions with group differences and clinical data after applying correction for multiple comparisons.

## Discussion

**Figure 3:**
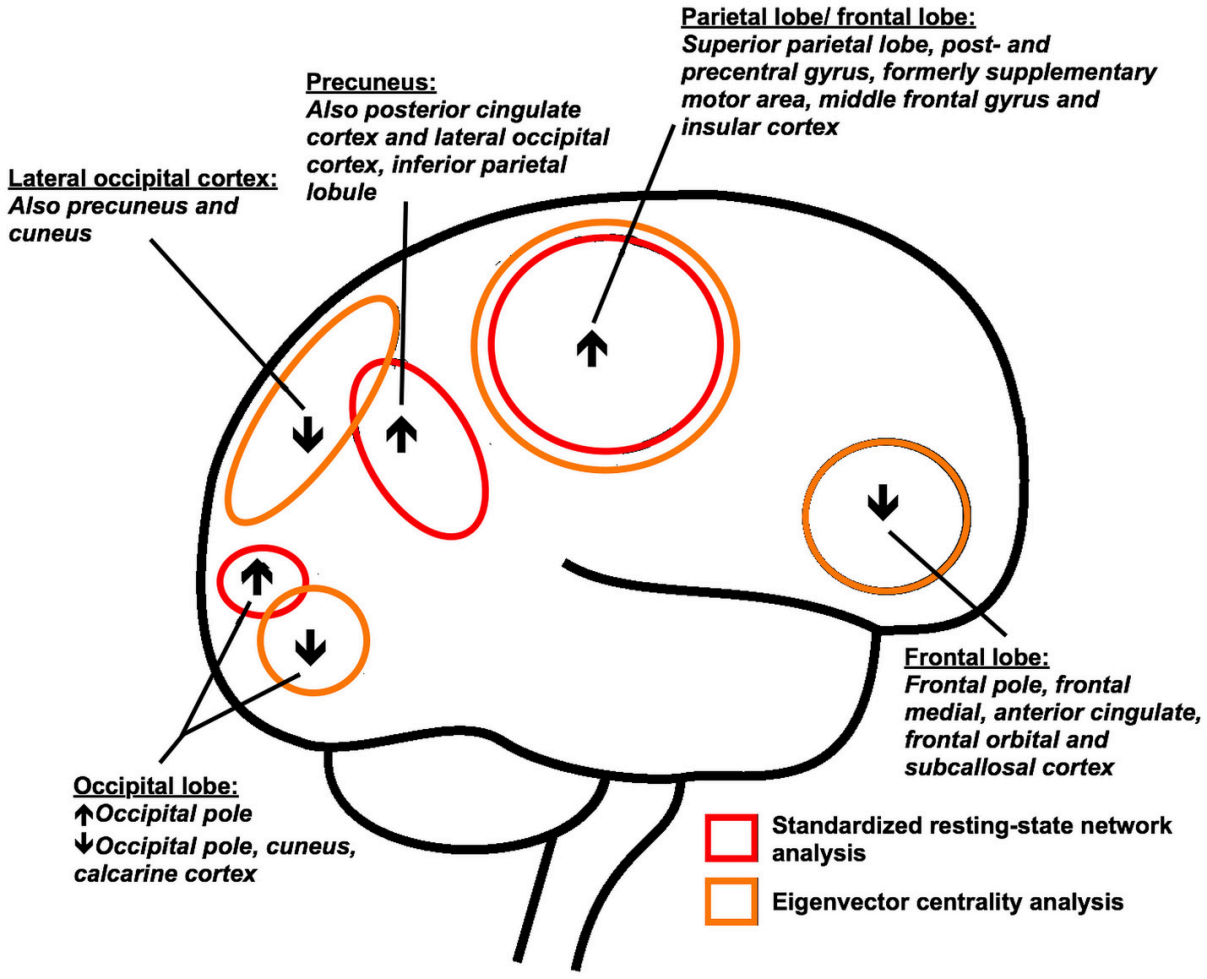
Main cortical differences (eigenvector centrality mapping and standard resting-state network analysis results) in resting-state functional connectivity between Parkinson's disease patients and control subjects are shown. Orange: whole-brain functional connectivity differences as measured with eigenvector centrality mapping; Red: functional connectivity differences in relation to eight standard resting-state networks; ↑: increased functional connectivity; ↓: decreased functional connectivity.

We used two complementary methods to analyze resting-state brain functional connectivity to study the cerebral functional reorganization at a network and whole brain level in patients with PD. Using the novel graph analysis approach (ECM), we found that frontoparietal regions display a stronger connectivity to the whole-brain network function in PD patients compared to control subjects, while a decreased connectivity was found for frontal and occipital areas of the brain (Figures 1, 3). In the resting-state networks of the brain, we found predominantly increased functional connectivity within the sensorimotor system and visual networks (Figures 2, 3). Comparing both approaches highlights a partial overlap of regional alterations of the whole brain functional connectivity architecture in PD (Figure 3).

### Regions of increased functional connectivity in Parkinson's disease

Mean EC values were increased in large frontoparietal brain regions of PD patients (Figures 1, 3), indicating that these regions fulfill a more prominent role within the overall brain network of PD patients. The method used in our study counts the neighbors of each vertex, weighted by their centralities, while prior studies often used degree centrality in PD, another graph analysis technique that estimates the number of connections of a node. These studies showed higher frontoparietal connectivity in patients compared to control subjects (7, 35). It was further found that the efficiency among these connections was increased in PD patients (35). The frontoparietal regions identified with ECM partly overlapped with increased regional functional connectivity identified by standard resting-state network analysis within the sensorimotor system network, including the sensorimotor, primary motor and premotor cortex, and SMA (Figures 1–3). Increased resting-state functional connectivity of regions involved in motor control is a consistent finding in treated PD patients, which may suggest a role of dopaminergic medication (7, 36–39). Alternatively, the findings may reflect compensatory reorganization of basal ganglia thalamo-cortical motor loops (40, 41), since increased resting-state functional connectivity in frontoparietal regions has been described in PD patients in the “off-medication-state” as well (35).

A recurrent finding in this study, identified with both approaches, was increased functional connectivity of the precuneus and posterior cingulate cortex in PD patients (Figures 1–3). Both brain structures are considered as highly connected and are key nodes in the DMN (31, 42, 43). Disruption of the DMN seems to be associated with cognitive deficits in PD patients (8). A recent study further observed increased connectivity between the precuneus and frontoparietal regions during task conditions, suggesting that the precuneus simultaneously interacts with multiple brain networks depending on the cognitive status (6, 43). However, the association of functional connectivity within the DMN with cognitive performance in the present study did not survive correction for multiple comparisons.

### Regions of decreased functional connectivity in Parkinson's disease

Occipital and frontal parts of the brain showed reduced EC in PD patients, indicating a diminished role of these regions within the overall brain functional architecture network in PD. Only two other studies applied whole brain ECM in PD. The first study aimed to investigate EC in depressed PD patients and also reported small frontoparietal regions of decreased EC in the non-depressed PD group scanned in the “off-medication-state,” compared to control subjects (10). The second study investigated ECM changes in PD patients following surgery, and reported that penetration of electrodes in the subthalamic nucleus was associated with increased EC in the brainstem, which was related to motor improvement (44). However, this is the first study in which whole brain ECM results of mild to moderate PD patients are compared to healthy control subjects. Resting-state fMRI studies using other techniques than ECM in PD, report connectivity reductions of frontal and occipital regions as well (7, 45), possibly related to cognitive dysfunction (46, 47).

In Alzheimer's disease, ECM shows decreased EC in the occipital cortex, but increased EC in frontal brain areas (28). Decreased occipital EC was associated with a poorer cognitive performance in Alzheimer's disease patients, as well as in control subjects (28). In PD, decreased EC of the occipital cortex of patients may further be related to disruption of visual networks (7, 39). Distinct spatial regions of the visual system that were identified with ECM, showed increased intra-network connectivity in PD patients within standard resting-state visual networks in this study (Figures 2, 3). Collectively, these findings suggest disrupted visual network organization and integration, which could be related to visual deficits or visuo-spatial attention deficits (31, 47, 48).

### Spatial distribution of altered functional connectivity

The regions of altered functional connectivity identified with two complementary techniques in this study are shown in Figure 3. Post mortem studies show that neocortical deposition of Lewy pathology occur in the temporo-occipital, temporal and frontal gyrus and the cingulate, insular and inferior parietal cortex (1, 4). We found altered functional connectivity in these structures with known Lewy pathology, but predominant regions of altered functional connectivity were located posterior in the brain, in particular the precuneus and posterior cingulate cortex. If the regionally altered functional connectivity in PD reflects the selective vulnerability of neuronal regions of the brain to α-SynA, cannot be assessed from this study. However, it is of interest that it was shown that the majority of α-SynA are not localized in Lewy bodies, but are in the form of much smaller aggregates than Lewy bodies (5, 49). These α-SynA were detected throughout the cortex of Lewy body disease patients and were most dense in the cingulate cortex (5, 49). In PD, smaller α-SynA than Lewy bodies can also be observed at predilection sites (5, 49).

The identified posterior brain regions further showed highest mean EC values in healthy control subjects, which is in line with findings of other studies reporting these regions as highly connected (hub) regions (28, 42, 50). In Alzheimer's disease patients, positon emission tomography amyloid imaging showed high levels of amyloid-β deposition in cortical hubs compared to control subjects (50). It is proposed that cortical hub regions display a preferential vulnerability to pathology in neurodegenerative diseases, due to their intrinsic high activity level, or due to the possibility that increased neuronal activity enhances the process of misfolded protein in the brain (3, 51–53). Non-invasive imaging of α-SynA has proven more challenging than imaging amyloid-β deposition, making it difficult to study the presence and density of α-SynA levels in specific cortical (hub) regions and test these assumptions in PD.

### Increased functional connectivity: pathogenic and compensatory mechanisms

A prominent finding in this study is the increased functional connectivity in PD patients. Increased functional connectivity is a common finding in neurodegenerative diseases (52, 54), but the mechanism underlying increased functional connectivity is unclear. It may simply reflect the primary disease process, or appear secondary in response to altered function elsewhere in the brain (55). The latter is often explained as a compensation mechanism in fMRI resting-state and task-based studies that found increased connectivity associated with lower symptom scores (52, 54). In PD, for instance, there is evidence for increased functional connectivity between the putamen and the cerebellum in mild to moderate stages of the disease, correlating with better motor performance (56). In the presence of manifested disease, as in our patient group, increased connectivity can indicate unsuccessful or only partial compensation (52) In this study, we found an altered balance of functional brain connectivity in symptomatic stages of PD in which α-SynA pathology involves neocortical areas (1, 4). Longitudinal fMRI studies are needed to assess if assumingly preserved neuronal areas show increased connectivity early in the disease process, if connectivity in these regions changes as the disease progresses, and if this is related to network levels of clinical performance.

## Potential limitations

Patients were scanned while taking their usual medications in this study. A potential modulatory role of dopaminergic medication cannot be ruled out. Several fMRI studies have reported a normalizing effect of dopaminergic medication on connectivity in PD, especially of sensorimotor regions, which might suggest that our findings would have been more pronounced if patients were scanned in the “off-medication-state” (57). Moreover, dopaminergic medication may lead to prolonged motor responses and its chronic use may alter brain organization (58, 59). Hence, if scanning PD patients after a certain period of withdrawal of dopaminergic drugs solely measures the influence of the disease process, is still open for debate. Further, scanning patients in the “on-medication-state” may reduce motion-related artifacts, which are especially problematic in resting-state fMRI studies. To reduce motion-related artifacts, we additionally visually inspected the data, performed standard motion correction and included white matter and cerebrospinal fluid templates in the analyses (21, 25, 26).

After strict correction for multiple comparisons, we found no relationship between functional connectivity and severity of motor symptoms (MDS-UPDRS motor scale), predominantly non-motor (non-dopaminergic) symptoms (SENS-PD scale) and cognition (SCOPA-COG scale) in PD. Dopaminergic medication may have attenuated the examined associations through a decrease of symptom scores that are responsive to dopaminergic stimulation and through a possible normalizing effect on functional connectivity in PD (57). However, we used LED as an additional covariate in our model and this did not alter the significance of our findings. Moreover, no relations were found between functional connectivity measures and clinical measures. Previous studies in PD using structural imaging methods used to study brain atrophy or changes of white matter tracts (fractional anisotropy or mean diffusivity), however have revealed moderate clinico-imaging relationships (6, 60, 61). This likely suggests that changes of the functional connectivity architecture of the brain provide a different perspective of the consequences of the pathobiology of PD, which do not necessarily have to be associated with clinical measures. This assumption is in line with studies in other neurodegenerative diseases, such as Alzheimer's disease, progressive nuclear palsy and Huntington's disease, where no clear associations have yet emerged between measures of functional connectivity and clinical measures of disease severity (6).

## Conclusions

The findings in this study on functional brain network organization in PD indicate a diminished role of frontal and occipital brain areas, while posterior parietal and frontoparietal areas display a more prominent connectivity to the whole-brain network function. We did not find significant correlations between clinical measures and functional imaging parameters, suggesting that changes on the level of functional connectivity architecture of the brain provide a different perspective of the pathological consequences of PD. Regionally altered functional connectivity was most pronounced in highly connected (hub) regions, particularly the posterior cingulate cortex and precuneus, which may account for the distributed abnormalities across the whole brain network architecture in PD.

## Ethics statement

This study was carried out in accordance with the recommendations of the Medical Ethics Committee of the LUMC. The protocol was approved by the Medical Ethics Committee of the LUMC. All subjects were informed about the objectives and demands of the study and signed the consent form in accordance with the Declaration of Helsinki.

## Author contributions

LdS composed the manuscript. LdS, JvdG, JM, JH, and JvH: recruited data. LdS and AH performed data analysis and visualization. AH, JvdG, JM, JH and JvH provided review and critique on the manuscript. JM and JvH conceptualization of the study.

### Conflict of interest statement

The authors declare that the research was conducted in the absence of any commercial or financial relationships that could be construed as a potential conflict of interest.

## Abbreviations

α-SynA: α-synuclein aggregates
ECM: eigenvector centrality mapping
MDS-UPDRS: Movement Disorder Society Unified Parkinson's Disease Rating Scale
SENS-PD: SEverity of Non-dopaminergic Symptoms in Parkinson's disease
SCOPA-COG: SCales for Outcomes in PArkinson's disease-COGnition
LDE: levodopa dose equivalent
DA: dopamine agonists.
